# Prevalence and genetic evolution analysis of porcine epidemic diarrhea virus and porcine circovirus type 2 in Sichuan Province, China, from 2023 to 2024

**DOI:** 10.3389/fvets.2024.1475347

**Published:** 2024-10-30

**Authors:** Fang Wu, Tong Xu, Si-Yuan Lai, Yan-Ru Ai, Yuan-Cheng Zhou, Liang-Peng Ge, Jing Sun, Zuo-Hua Liu, Xiu Zeng, Li-Qiao Lang, Zhi-Wen Xu, Ling Zhu

**Affiliations:** ^1^College of Veterinary Medicine, Sichuan Agricultural University, Chengdu, China; ^2^Key Laboratory of Animal Breeding and Genetics, Key Laboratory of Sichuan Province, Sichuan Animal Science Academy, Chengdu, China; ^3^Livestock and Poultry Biological Products, Key Laboratory of Sichuan Province, Sichuan Animal Science Academy, Chengdu, China; ^4^National Center of Technology Innovation for Pigs, Chongqing Academy of Animal Sciences, Chongqing, China; ^5^College of Veterinary Medicine Sichuan, Key Laboratory of Animal Epidemic Disease and Human Health, Sichuan Agricultural University, Chengdu, China

**Keywords:** porcine circovirus type 2, porcine epidemic diarrhea virus, epidemiological survey, genetic evolution analysis, coinfection

## Abstract

**Introduction:**

Porcine circovirus type 2 (PCV2) and Porcine epidemic diarrhea virus (PEDV) are highly prevalent in Sichuan, significantly affecting the swine industry’s development. PCV2, known for its immunosuppressive effects, can compromise pigs’ immune systems, while PEDV typically causes diarrhea in piglets, leading to high mortality rates. Despite their impact, recent studies on the epidemiology and genetic diversity of PCV2 and PEDV within Sichuan Province remain limited.

**Methods:**

This study examines clinical samples from 352 diarrheal piglets across 63 pig farms in 17 regions of Sichuan Province, revealing positivity rates of 42.33% (149/352) for PCV2 and 50.28% (177/352) for PEDV, with a co-infection rate of 27.56% (97/352). Notably, the highest positivity rates were observed in Ziyang for PCV2 at 61.90% (13/21), and in Meishan for PEDV at 73.81% (31/42), both regions also reported the highest co-infection rates of 47.62%.

**Results and discussion:**

Seasonal analysis indicated that PEDV infections peaked during winter, whereas PCV2 showed no significant seasonal trends. Phylogenetic analysis identified 14 PCV2 strains, categorizing 2 as PCV2b (14.29%), 10 as PCV2d (71.43%), and 2 as PCV2e (14.29%). Among the 16 PEDV strains, 2 were classified as G1a (12.5%) and 14 as G2a (87.5%), with PCV2d and PEDV G2a identified as the predominant strains in the region. The study also highlights a high mutation rate at the antigenic sites of both viruses, potentially affecting vaccine efficacy. These findings underscore the need for ongoing surveillance and vaccine development tailored to the prevalent strains to improve control measures within the province.

## 1 Introduction

Porcine Circovirus Type 2 (PCV2), classified within the Circovirus genus of the Circoviridae family, is known as the smallest non-enveloped animal virus (1). It is characterized by a circular single-stranded DNA genome approximately 1.76 kb in size (2). The genome is structured into two primary open reading frames (ORFs): ORF1 and ORF2. ORF1 encodes two non-structural proteins, Rep and Rep’, which are integral to the virus’s replication and regulation, and are notably conserved across various strains (3). ORF2, on the other hand, encodes the capsid protein (Cap), which is essential for viral entry and the stimulation of virus-neutralizing antibodies, and exhibits a higher mutation rate (4). PCV2 is identified as the most dominant and impactful pathogen among the Porcine Circovirus Associated Diseases (PCVAD), which include Post-Weaning Multisystemic Wasting Syndrome (PMWS), Porcine Dermatitis and Nephropathy Syndrome (PDNS), interstitial pneumonia, and reproductive disorders (5). Additionally, PCV2 is associated with enteritis, initially presenting as pale-yellow diarrhea that eventually turns black as the infection progresses (6).

PCV2 was first identified in the late 1990s in Canada and the United States, with PCV2a being the initial genotype. As we moved into the 2000s, PCV2b started to emerge as the dominant genotype globally, replacing PCV2a. This shift underscored the dynamic nature of viral evolution and its impact on disease management in swine populations. By the 2010s, another shift occurred with the emergence of PCV2d, which has since been reported as the prevalent genotype in several countries, including China (7).

Porcine Epidemic Diarrhea Virus (PEDV), a member of the Alphacoronavirus genus within the Coronaviridae family, is an enveloped entity featuring a single-stranded, positive-sense RNA genome of approximately 28 kb in length (8). This genomic structure includes seven open reading frames (ORFs). At the 5’end, ORF1a and ORF1b encode two polyproteins, pp1a and pp1b, essential for viral replication. At the 3’end, five additional ORFs encode four structural proteins--spike (S), envelope (E), membrane (M), and nucleocapsid (N) proteins--and one accessory protein (9). Notably, the S gene exhibits significant genetic diversity, particularly within the S1 domain, which is critical for altering PEDV virulence and adapting the virus to *in vitro* growth conditions (10). The S protein, a trimeric glycoprotein on the virus’s surface, is instrumental for attachment, receptor binding, and cell membrane fusion during viral entry, and contains most of the neutralizing epitopes, including the CO-26 K equivalent (COE) domain (11), SS2, SS6 (12), and 2C10, which are the primary targets for vaccine development against PEDV (13).

Porcine Epidemic Diarrhea (PED), caused by PEDV, is characterized by severe symptoms including diarrhea, vomiting, anorexia, and dehydration, impacting pigs of all ages but is particularly lethal to piglets less than 7 days old (14). PEDV, first detected in the late 1970s, has had a long history of circulation, particularly in Europe (15). Despite the widespread use of the CV777 vaccine, China faced a severe PEDV outbreak in 2010 (16), which was attributed to the emergence of non-S INDEL strains (17). This outbreak highlighted the challenges of controlling viral diseases even with vaccination strategies in place. Later, new S INDEL strains were also identified within China (18), indicating ongoing viral evolution and the need for continuous surveillance and vaccine adaptation.

Previous studies have reported regional trends in PCV2 and PEDV prevalence. A survey conducted in Sichuan Province showed that PCV2 positivity rates increased over the years, from 24.14% in 2020 to 29.85% in 2022. Among the 13 PCV2 strains collected, 1 strain belonged to the PCV2a genotype, 5 strains to PCV2b, and 7 strains to PCV2d, indicating that PCV2d is the predominant genotype in the region (19). Similarly, research on PEDV in Sichuan Province identified the GIIa genotype group as the most prevalent. Among the 10 collected PEDV sequences, 1 strain was classified as GIa, 8 strains as GIIa, and 1 strain as GIIb (13). Additionally, a broader study conducted between 2014 and 2018 in both Sichuan and Guizhou provinces reported a PEDV positive rate of 35.81%. The strains were categorized into subgroups, with the S-INDEL (G1c) accounting for 15.38%, and the non-S-INDEL subgroups (G2b, AJ1102-like, and G2c) comprising 23.08 and 59.62%, respectively (17).

Porcine Circovirus Type 2 (PCV2) is universally found in pig farms, leading to significant immunosuppression upon infection (20). The clinical manifestations, including symptoms and organ lesions, typically result from the synergistic effects of co-infections that amplify pathogenicity and complicate the management of diseases (21). For example, co-infection with PCV2 and Porcine Reproductive and Respiratory Syndrome Virus (PRRSV) not only exacerbates immunosuppression but also modifies the host’s defense mechanisms, heightening the risk of secondary bacterial and viral infections (22). Similarly, pigs suffering from concurrent PCV2 and Porcine Parvovirus (PPV) infections experience more severe illness and damage than those infected with PCV2 alone (23). A study showed that out of 107 small intestine samples from pigs naturally infected with PEDV, 35 samples (32.7%) tested positive for PCV2 (24). In piglets infected with PCV2, PEDV infection led to more severe diarrhea (5). Despite the prevalent co-occurrence of PCV2 and Porcine Epidemic Diarrhea Virus (PEDV) within China’s pig populations, investigations into their co-infection dynamics are limited. This study explores the co-infection rates of PCV2 and PEDV in piglets presenting with severe diarrheal symptoms in Sichuan, China. Furthermore, an in-depth analysis of the genetic variations in PCV2 and PEDV has been undertaken, highlighting the complexity of managing diseases within co-infected populations.

## 2 Materials and methods

### 2.1 Sample collection and processing

**Figure 1:**
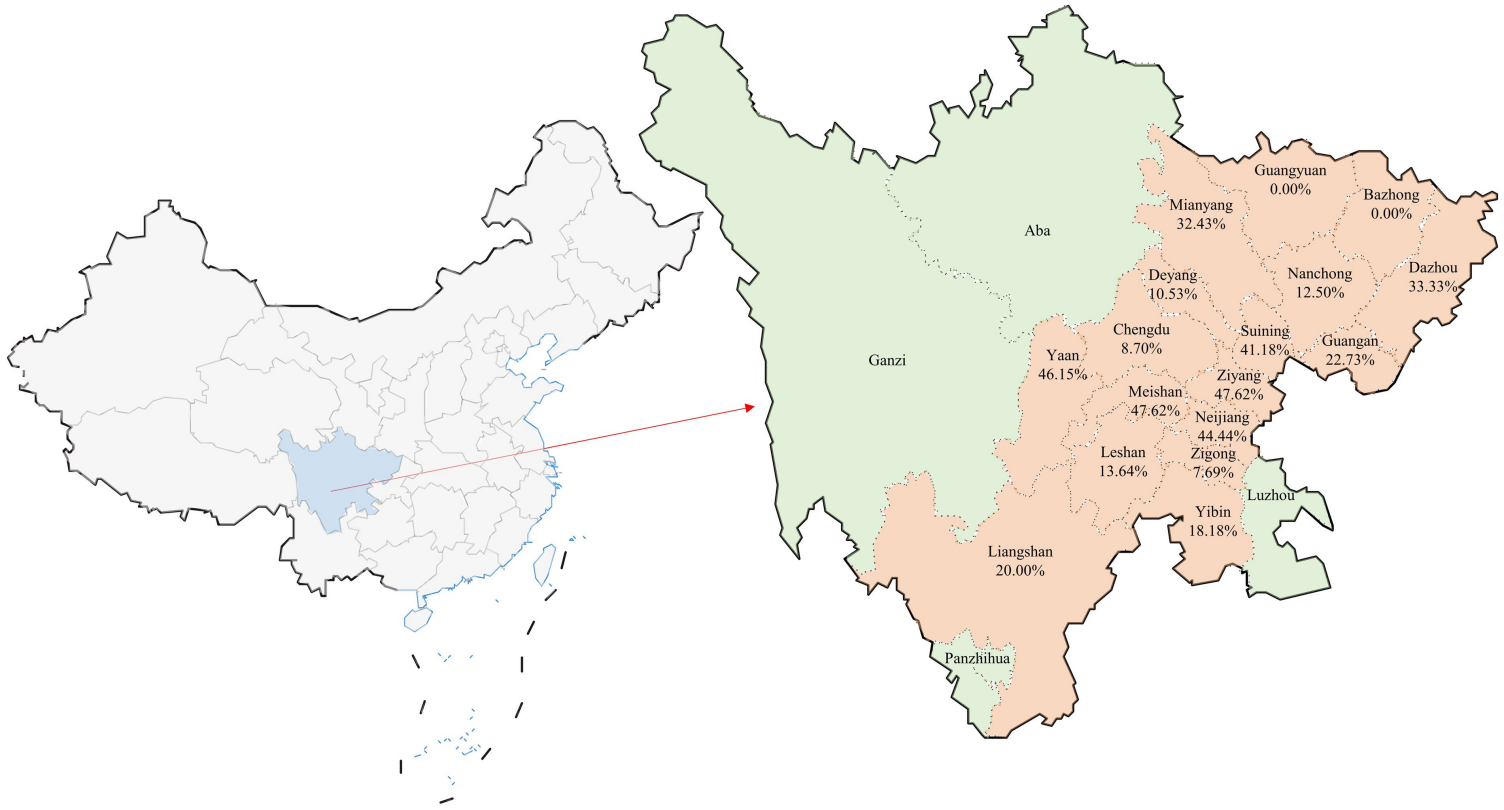
Geographic distribution of clinical sample collection and co-infection rates in Sichuan Province, China. The figure provides a visual representation of the distribution of the 352 clinical samples collected from Sichuan Province, China. This map illustrates the cities involved in the study with the specific focus on the co-infection rates of Porcine Circovirus Type 2 (PCV2) and Porcine Epidemic Diarrhea Virus (PEDV). Cities that did not participate in the sample collection are highlighted in green. Below each city name included in the study, the percentage indicates the observed co-infection rate of PCV2 and PEDV, providing a clear overview of the regional variations in co-infection prevalence across the province. This visual aids in understanding the geographic spread and impact of these infections within the surveyed areas.

Between January 2023 and July 2024, a total of 352 small intestine samples were gathered from diarrheal piglets distributed across 63 pig farms located in 17 cities within Sichuan Province. These cities included Guangyuan, Bazhong, Dazhou, Mianyang, Nanchong, Guang’an, Deyang, Suining, Chengdu, Ziyang, Ya’an, Meishan, Neijiang, Leshan, Zigong, Yibin, and Liangshan (Figure 1).

In this study, a stratified random sampling method was employed. First, the number of samples was proportionally allocated based on the number and density of pig farms in the 17 districts (Table 1). Then, pig farms were randomly selected within each district for sample collection. To dynamically adjust the sample size, the specific number of samples was determined according to the size of each pig farm. Within each farm, piglets exhibiting symptoms of diarrhea were prioritized for preliminary screening and served as the candidate group. To ensure randomness and scientific rigor in the sampling process, these diarrheal piglets were systematically numbered using an identification method. The appropriate sampling ratio was determined based on the size of the pig farm and the number of diarrheal piglets, and samples were selected from the numbered group through systematic random sampling at fixed intervals. All samples were collected by professionally trained technicians following aseptic operation standards to ensure proper procedure.

**Table 1:** Infection and co-infection rates of PCV2 and PEDV in various cities of Sichuan Province, 2023–2024.

Cities	PCV2	PEDV	Co-infection
Guangyuan	5/15 (33.33%)	7/15 (46.67%)	0/15 (0.00%)
Bazhong	2/12 (16.67%)	5/12 (41.67%)	0/12 (0.00%)
Dazhou	12/21 (57.14%)	10/21 (47.62%)	7/21 (33.33%)
Mianyang	18/37 (48.65%)	21/37 (56.76%)	12/37 (32.43%)
Nanchong	6/24 (25.00%)	5/24 (20.83%)	3/24 (12.50%)
Guangan	9/22 (40.91%)	10/22 (45.45%)	5/22 (22.73%)
Deyang	4/19 (21.05%)	12/19 (63.16%)	2/19 (10.53%)
Suining	8/17 (47.06%)	11/17 (64.71%)	7/17 (41.18%)
Chengdu	5/23 (21.74%)	9/23 (39.13%)	2/23 (8.70%)
Ziyang	13/21 (61.90%)	12/21 (57.14%)	10/21 (47.62%)
Yaan	21/39 (53.85%)	19/39 (48.72%)	18/39 (46.15%)
Meishan	23/42 (54.76%)	31/42 (73.81%)	20/42 (47.62%)
Neijiang	5/9 (55.56%)	5/9 (55.56%)	4/9 (44.44%)
Leshan	9/22 (40.91%)	4/22 (18.18%)	3/22 (13.64%)
Zigong	3/13 (23.08%)	6/13 (46.15%)	1/13 (7.69%)
Yibin	4/11 (36.36%)	7/11 (63.64%)	2/11 (18.18%)
Liangshan	2/5 (40.00%)	3/5 (60.00%)	1/5 (20.00%)
Total	149/352 (42.33%)	177/352 (50.28%)	97/352 (27.56%)

The collected samples were placed in sterile 1.5 mL centrifuge tubes containing sterile PBS, homogenized, and subjected to three freeze–thaw cycles. Subsequently, the samples were centrifuged at 12,000 × g to separate the supernatant. Nucleic acids were extracted from the supernatant using the FastPure Viral DNA/RNA Mini Kit (Vazyme, Nanjing, China), and reverse transcription was carried out using PrimeScript™ RT Master Mix (Takara, Dalian, China). The extracted nucleic acids and the resultant cDNA were then stored at −80°C, prepared for further experimental analysis.

### 2.2 Sequencing of the PCV2 whole genomes and PEDV S genomes

To detect PCV2 and PEDV in clinical samples, a TB green II-based real-time fluorescent quantitative PCR was employed for PCV2 (25). The forward primer is 5’-CAACGGAGTGACCTGTCT-3′, and the reverse primer is 5’-ACCATCCCACCACTTGTT-3′. The standard curve equation is y = −3.4486x + 39.533. Meanwhile, a SYBR green I-based real-time fluorescent quantitative PCR was utilized for PEDV (26). The forward primer is 5’-AAATGGGAAGTCGGCAGA-3′, and the reverse primer is 5’-GTTTTGTTGTGGCGGTAG-3′. The standard curve equation for PEDV is y = −3.4589x + 37.455. Following the protocols previously described (3, 10), sequencing was performed on samples that tested positive to obtain the full genome sequence of PCV2 and the S gene sequence of PEDV for phylogenetic analysis. The forward primer for PCV2 is 5’-GAC CGC GGG CTG GCT GAA CTTT TGA AAG T − 3′, and the reverse primer is 5’-GAC CCG CGG AAA TTT CTG A CAA ACG TTACA-3′. The PEDV S gene is sequenced in two segments. The forward primer for the first segment is 5’-AGTGGCGCTGTGATTGA-3′, and the reverse primer is 5’-TAGGCGTGCCAGTAATCAAC-3′. The forward primer for the second segment is 5’-TGATTACTGGCACGCCTAAA-3′, and the reverse primer is 5’-CCGTCACATTTGAAGCTTGTC-3′.

The amplified PCR products were purified and subsequently ligated into the pMD19-T cloning vector (Takara, Beijing, China). These expression vectors were transformed into DH5α competent cells (Takara, Beijing, China) to facilitate propagation. The expression clones were forwarded to Sangon Biotech Co., Ltd. (Shanghai, China) for sequencing. Using the EditSeq and MegAlign tools within the Lasergene software package (DNAStar, Inc., Madison, WI), the segmented sequences of PCV2 and PEDV were assembled into complete sequences, respectively, enabling detailed genetic analysis.

### 2.3 Sequence analyses

The complete genomic sequences of PCV2 and the S gene sequences of PEDV obtained in this study, along with reference sequences sourced from GenBank (Table 2; Table 3). The ORF2 sequences of PCV2 strains and the S gene sequences of PEDV were aligned using the MegAlign program within the Lasergene software package. Phylogenetic analyses were conducted using the Molecular Evolutionary Genetics Analysis (MEGA11) software, employing the Maximum Likelihood method. This method utilized the HKY + G model for PCV2 and included 1,000 bootstrap replications. Similarly, the GTR + G + I model was applied for PEDV, also with 1,000 bootstrap replications.

**Table 2:** Sample information and GenBank accession numbers of PCV2 reference sequences and detection sequences.

Virus strain	Countries	Collection year	GenBank accession no.	genotype
pmws PCV	Canada	1997	AF027217	PCV2a
PCV2	United Kingdom	1998	AF055392	PCV2a
LG	China	2008	HM038034	PCV2a
10JS-2	China	2010	JQ806749	PCV2a
PCV2	China	2002	AY177626	PCV2b
NB0301	China	2003	AY391729	PCV2b
putian0401	China	2005	DQ180393	PCV2b
DBN-SX07-2	China	2007	HM641752	PCV2b
DK1980PMWSfree	Denmark	2007	EU148503	PCV2c
DK1987PMWSfree	Denmark	2007	EU148504	PCV2c
DK1990PMWSfree	Denmark	2007	EU148505	PCV2c
SH	China	2004	AY686763	PCV2d
TJ06	China	2007	EF524539	PCV2d
BJ0901b	China	2009	GU001710	PCV2d
KU-1607	South Korea	2016	KX828234	PCV2d
GX0601	China	2007	EF524532	PCV2e
HB0602	China	2007	EF524537	PCV2e
BJ0901a	China	2009	GU001709	PCV2e
YN-8	China	2009	HM776452	PCV2f
1,314-09-1	Croatia	2009	HQ591381	PCV2f
LN6/1999	China	1999	MF278777	PCV2f
P2425NT	Vietnam	2008	JX099786	PCV2g
ZrBd_wb UKR	Poland	2010	KP420197	PCV2g
BG0-1	Vietnam	2011	JQ181592	PCV2h
NAVET_vietnam3	Vietnam	2004	JX506730	PCV2h
549-QNa	Vietnam	2009	KM042398	PCV2h
SCABTC-PCV2-DW	China	2023	PQ123983	PCV2d
SCABTC-PCV2-FEI	China	2024	PQ123984	PCV2d
SCABTC-PCV2-HT	China	2023	PQ123985	PCV2b
SCABTC-PCV2-JLQ	China	2023	PQ123986	PCV2d
SCABTC-PCV2-NJA	China	2023	PQ123987	PCV2d
SCABTC-PCV2-NJB	China	2023	PQ123988	PCV2d
SCABTC-PCV2-PJA	China	2024	PQ123989	PCV2e
SCABTC-PCV2-PJB	China	2024	PQ123990	PCV2e
SCABTC-PCV2-PJG	China	2024	PQ123991	PCV2d
SCABTC-PCV2-S	China	2023	PQ123992	PCV2b
SCABTC-PCV2-SY	China	2023	PQ123993	PCV2d
SCABTC-PCV2-XWD	China	2023	PQ123994	PCV2d
SCABTC-PCV2-YQL	China	2024	PQ123995	PCV2d
SCABTC-PCV2-ZB	China	2024	PQ123996	PCV2d

**Table 3:** Sample information and GenBank accession numbers of PEDV S gene reference sequences and detection sequences.

Virus strain	Countries	Collection year	GenBank accession no.	genotype
CV777	Switzerland	2001	AF353511	G1a
CH/S	China	2011	JN547228	G1a
virulent DR13	South Korea	2009	JQ023161	G1a
CH9-FJ	China	2011	JQ979287	G1a
CH13-GX	China	2011	JQ979288	G1a
CH22-JS	China	2011	JQ979290	G1a
CH/YNKM/2012	China	2012	JX018180	G1a
SD-M	china	2012	JX560761	G1a
JS2008	China	2012	KC109141	G1a
OH851	United States	2014	KJ399978	G1b
USA/Indiana12.83/2013	United States	2013	KJ645635	G1b
USA/Minnesota58/2013	United States	2013	KJ645655	G1b
USA/Ohio126/2014	United States	2014	KJ645702	G1b
KNU-1406-1	South Korea	2014	KM403155	G1b
15 V010/BEL/2015	Belgium	2015	KR003452	G1b
NL/GD001/2014	Netherlands	2014	KR011121	G1b
CH-YGC-01-2015	China	2015	KR296678	G1b
ZL29	China	2015	KU847996	G1b
L00721/GER/2014	Germany	2014	LM645057	G1b
CH/ZMDZY/11	China	2011	KC196276	G2a
KNU-1401	South Korea	2014	KJ451047	G2a
USA/Iowa28/2013	United States	2013	KJ645636	G2a
USA/Kansas29/2013	United States	2013	KJ645637	G2a
USA/Texas128/2013	United States	2013	KJ645697	G2a
PEDV-LYG	China	2014	KM609212	G2a
PEDV-WS	China	2014	KM609213	G2a
CH/HNAY/2015	China	2015	KR809885	G2a
YC2014	China	2014	KU252649	G2a
JSCZ1601	China	2016	KY070587	G2a
HBJZ2	China	2016	KY775039	G2a
HBXY1	China	2016	KY775040	G2a
ZJ-2011-2	China	2011	JN825711	G2b
BJ-2011-1	China	2011	JN825712	G2b
CH/YY/11	China	2011	JQ257006	G2b
CH17-GZ	China	2011	JQ979289	G2b
CH18-Hainan	China	2011	JQ979291	G2b
CH/FJXM-1/2012	China	2012	JX070671	G2b
GD-A	China	2012	JX112709	G2b
BJ-2012-2	China	2012	JX435300	G2b
HB-2012-2	China	2012	JX435303	G2b
HB-2012-4	China	2012	JX435305	G2b
LC	China	2011	JX489155	G2b
HLJ-2012	China	2012	JX512907	G2b
AH2012	China	2012	KC210145	G2b
HN6-201211	China	2012	KC242911	G2b
CH/JX-1/2013	China	2013	KF760557	G2b
CH/JX-2/2013	China	2013	KJ526096	G2b
VN/JFP1013_1/2013/Vinh An/Vietnam	Thailand	2013	KJ960178	G2b
VN/VAP1113_1/2013/Vung Tua/Vietnam	Thailand	2013	KJ960179	G2b
VN/KCHY-310113/2013/KhoaiChau/HungYen/Vietnam	Thailand	2013	KJ960180	G2b
FL2013	China	2013	KP765609	G2b
YN144	China	2014	KT021232	G2b
CH-SDLY-2-2014	China	2014	KU133254	G2b
CH-SDLY-3-2014	China	2014	KU133255	G2b
HBJM1	China	2016	KY775035	G2b
CH/HNYF/14	China	2014	KP890336	G2c
CH/HNQX-3/14	China	2015	KR095279	G2c
SCABTC-PEDV-CY	China	2023	PQ123997	G2a
SCABTC-PEDV-DK	China	2024	PQ123998	G2a
SCABTC-PEDV-HAI	China	2023	PQ123999	G1a
SCABTC-PEDV-HH	China	2023	PQ124000	G2a
SCABTC-PEDV-HT	China	2023	PQ124001	G2a
SCABTC-PEDV-JC	China	2023	PQ124002	G2a
SCABTC-PEDV-JL	China	2024	PQ124003	G2a
SCABTC-PEDV-M6	China	2024	PQ124004	G2a
SCABTC-PEDV-M7	China	2024	PQ124005	G2a
SCABTC-PEDV-M8	China	2024	PQ124006	G2a
SCABTC-PEDV-PG	China	2024	PQ124007	G1a
SCABTC-PEDV-SL	China	2023	PQ124008	G2a
SCABTC-PEDV-T	China	2023	PQ124009	G2a
SCABTC-PEDV-XN	China	2024	PQ124010	G2a
SCABTC-PEDV-ZY	China	2023	PQ124011	G2a
SCABTC-PEDV-ZYC	China	2023	PQ124012	G2a

Further, recombination detection was performed utilizing the integrated tools RDP, GENECONV, MaxChi, Chimaera, BootScan, SiScan, and 3Seq within the Recombination Detection Program (RDP 4.39). Recombination events that were corroborated by four or more programs were considered significant. For this analysis, the window size was set to 20 bp, and the maximum acceptable *p*-value was determined to be 0.01.

## 3 Results

### 3.1 Epidemic situation of PCV2 and PEDV in Sichuan Province, China, from 2023 to 2024

The analysis of 352 clinical samples revealed positivity rates of 42.33% (149/352) for PCV2 and 50.28% (177/352) for PEDV, with a co-infection rate of 27.56% (97/352). Examination of 63 pig farms showed that 61.90% (39/63) had positive cases of PCV2, 65.08% (41/63) for PEDV, and 34.92% (22/63) exhibited co-infections of both viruses. In the 17 cities surveyed, the positivity rates for PCV2, PEDV, and their co-infections varied significantly (Table 1):

- Guangyuan recorded rates of 33.33% for PCV2, 46.67% for PEDV, and no co-infections.
- Bazhong showed 16.67% for PCV2, 41.67% for PEDV, also with no co-infections.
- Dazhou had higher rates with 57.14% for PCV2, 47.62% for PEDV, and 33.33% for co-infections.
- In Mianyang, rates were 48.65% for PCV2, 56.76% for PEDV, and 32.43% for co-infections.
- Nanchong showed lower positivity with 25.00% for PCV2, 20.83% for PEDV, and 12.50% for co-infections.
- Guang’an had 40.91% for PCV2, 45.45% for PEDV, and 22.73% for co-infections.

Ziyang displayed the highest PCV2 positivity rate, whereas Meishan exhibited the highest PEDV positivity rate, and both cities recorded the highest rates of co-infections.

Seasonally, the infection rates for PCV2 were 24.83% in spring, 15.44% in summer, 23.49% in autumn, and 36.24% in winter. For PEDV, the corresponding rates were 24.29% in spring, 5.08% in summer, 12.43% in autumn, and 58.19% in winter. Co-infection rates mirrored this seasonal distribution with 26.80% in spring, 2.06% in summer, 19.59% in autumn, and 51.55% in winter.

### 3.2 PCV2 sequencing and phylogenetic analysis

In this investigation, we sequenced 14 full genomes of Porcine circovirus type 2 (PCV2) and 16 S genes of Porcine epidemic diarrhea virus (PEDV), subsequently uploading the sequences to GenBank (Table 2; Table 3). Within the PCV2 genomes analyzed, 12 were each 1767 base pairs (bp) in length. However, two strains, SCABTC-PCV2-PJA and SCABTC-PCV2-PJB, exhibited a thymine insertion at position 1,042, thereby extending their genome length to 1768 bp. All strains had a rep gene (ORF1) length of 945 bp, while the cap gene (ORF2) varied, being 702 bp in 2 strains and 705 bp in the remaining 12.

Homology analysis of the 14 PCV2 full genomes displayed a variability in genetic similarity, with homology ranging from 94.1 to 99.9%. For the ORF1 gene, homology ranged from 96.8 to 100%, and for the ORF2 gene, it varied from 88.6 to 99.7%. When compared to 25 reference strains, the homology of these isolates’ genomes ranged from 91.3 to 99.8%, with ORF1 gene homology between 96.5 to 99.5% and ORF2 gene homology between 81.3 to 99.7%.

At the protein level, the 14 PCV2 rep proteins demonstrated a homology of 98.4 to 100.0%, and the cap proteins varied from 85.5 to 100.0%. Relative to the Chinese vaccine strains AY686763 and HM641752, the homology of the rep protein was between 98.4 to 99.7%, and the cap protein ranged from 87.6 to 100.0%. This extensive comparison underscores the genetic variability within PCV2 populations.

We obtained complete genome sequences of 25 PCV2 reference strains from GenBank, encompassing genotypes PCV2a through PCV2h. The information on the PCV2 strains obtained in this experiment, along with the reference strains (Table 2). Given the high variability and antigenicity of the PCV2 Cap protein, it is commonly utilized in phylogenetic and amino acid analyses. In this study, we focused our phylogenetic analysis on the ORF2 gene. We compared the ORF2 genes of these 25 representative PCV2 strains with those of the 14 PCV2 sequences acquired during our investigation. The phylogenetic tree was constructed using the Maximum Likelihood method, employing the HKY + G model with 1,000 bootstrap replications, facilitated by the MEGA11 software.

**Figure 2:**
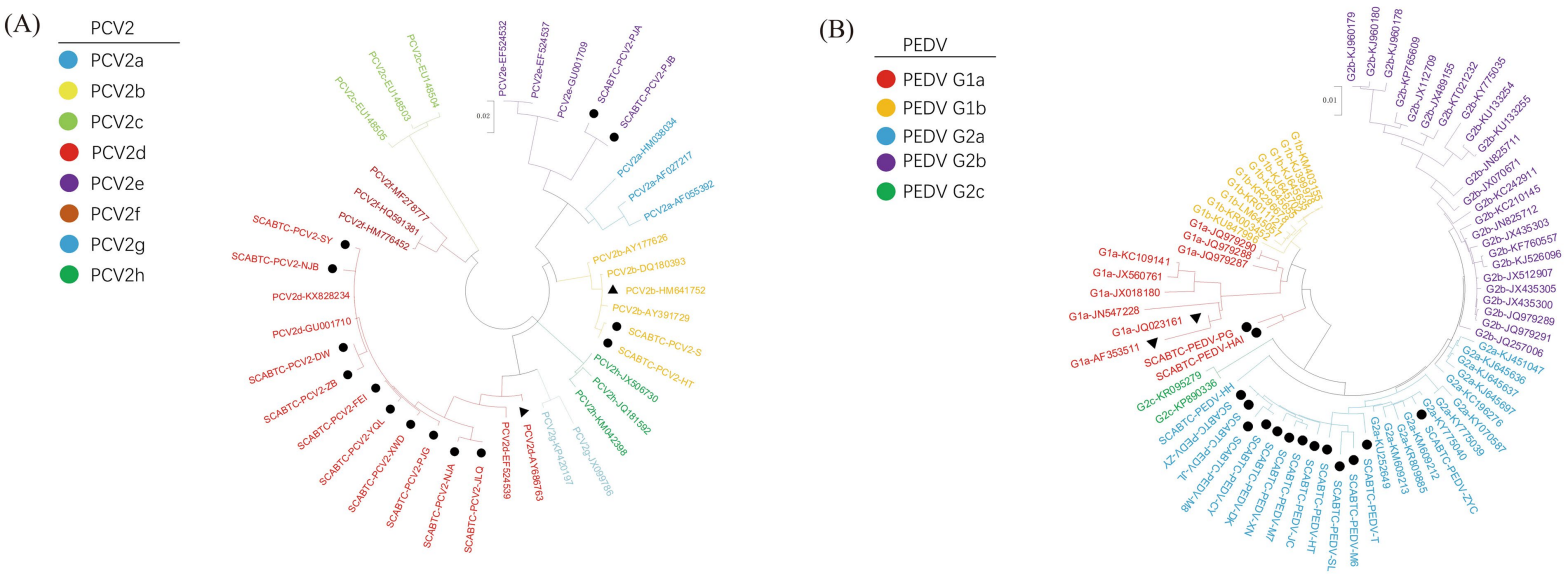
Phylogenetic analysis of PCV2 and PEDV strains. The figure presents two phylogenetic trees that illustrate the genetic relationships of strains based on their gene sequences: Picture A depicts the phylogenetic tree for Porcine Circovirus Type 2 (PCV2) based on the ORF2 gene, and Picture B shows the tree for Porcine Epidemic Diarrhea Virus (PEDV) based on the S gene. In both panels, strains that were obtained in this study are marked with solid circles to distinguish them from other strains. Vaccine strains, which are of particular interest due to their role in prevention strategies, are indicated by solid triangles.

The analysis revealed diverse genotypic distribution among the 14 sequences: 2 were identified as PCV2b (14.29%), 2 as PCV2e (14.29%), and the majority, 10 sequences, as PCV2d (71.43%) (Figure 2).

**Figure 3:**
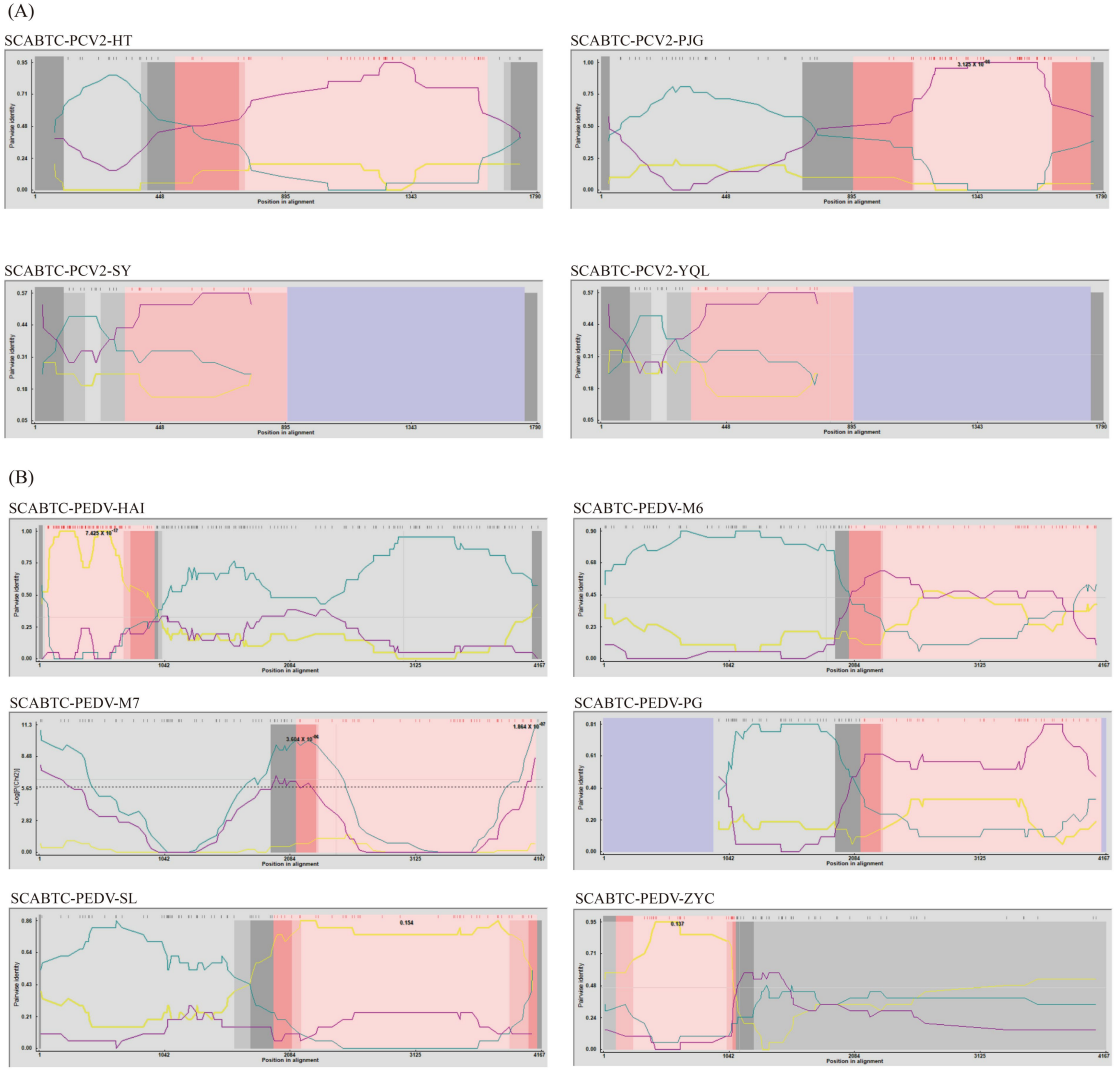
Recombination analysis of PCV2 and PEDV using RDP4. The figure displays the results of a recombination analysis for Porcine Circovirus Type 2 (PCV2) and Porcine Epidemic Diarrhea Virus (PEDV) conducted using the Recombination Detection Program version 4 (RDP4). Picture A of the figure is dedicated to PCV2, illustrating the recombination events detected within this virus, while Picture B focuses on PEDV, showing similar analysis results.

In this study, the 14 complete genome sequences of PCV2 obtained from the experiment, along with 25 reference sequences, were aligned for a comprehensive recombination analysis (Figure 3). Through this rigorous assessment, four recombination events were identified among the 14 complete PCV2 genome sequences (Table 4).

**Table 4:** Sequence recombination analysis.

Virus	Recombinant	Major parent	Minor parent	Breakpoint
PCV2	SCABTC-PCV2-HT	AY686763	AY177626	490–1,590 bp
	SCABTC-PCV2-PJG	HM038034	KX828324	889–1720 bp
	SCABTC-PCV2-YQL	KM042398	KX828324	310–888 bp
	SCABTC-PCV2-SY	KM042398	KX828324	310–888 bp
PEDV	SCABTC-PEDV-HAI	SCABTC-PEDV-JL	KJ960178	50–938 bp
	SCABTC-PEDV-M6	SCABTC-PEDV-SL	JX070671	2032–4,077 bp
	SCABTC-PEDV-M7	SCABTC-PEDV-SL	JX070671	2,125–4,110 bp
	SCABTC-PEDV-PG	SCABTC-PEDV-SL	JX070671	2,113–4,109 bp
	SCABTC-PEDV-SL	KU252649	JX435305	1938–4,119 bp
	SCABTC-PEDV-ZYC	JX435305	KJ645697	106–1,090 bp

One notable recombination event was detected in the SCABTC-PCV2-HT strain, where AY686763 served as the major parent and AY177626 as the minor parent, with the predicted breakpoint occurring between 490 bp and 1,590 bp. Another recombination event was observed in the SCABTC-PCV2-PJG strain, involving HM038034 as the major parent and KX828324 as the minor parent, with the breakpoint predicted between 889 bp and 1720 bp. Additionally, recombination events were detected in the SCABTC-PCV2-YQL and SCABTC-PCV2-SY strains, occurring between positions 310 bp and 888 bp, with KM042398 acting as the major parent and KX828324 as the minor parent.

According to the recombination analysis results, these recombination events are considered credible, adding significant insights into the genetic diversity of PCV2.

### 3.3 Comparative analysis of amino acid sequences of PCV2 cap protein

**Figure 4:**
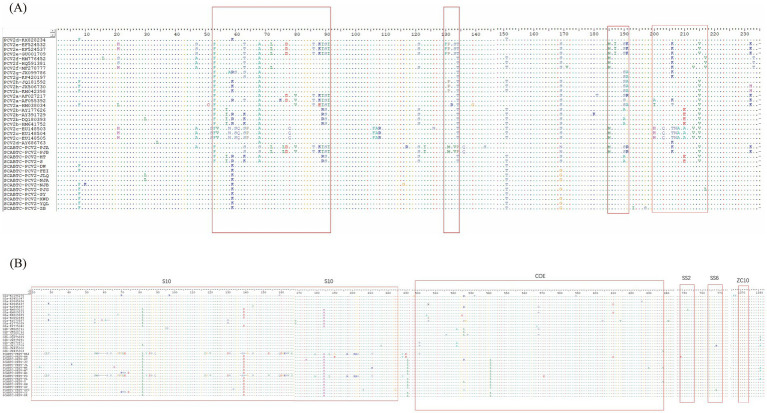
Amino acid analysis of PCV2 cap protein and PEDV S protein. The figure illustrates the amino acid analysis of two significant proteins in porcine viruses. Picture A displays the analysis for the PCV2 Cap protein, where highly variable regions within the protein structure are highlighted by red open boxes. Picture B shows the amino acid analysis for the PEDV S protein, with red open boxes identifying the five key antigenic sites: S10, COE, SS2, SS6, and ZC10.

This section presents a comparative analysis of the Cap protein amino acid sequences between the strains obtained in this study and reference strains, including the vaccine strains AY686763 and HM641752 (Figure 4). The PCV2b strains were characterized by specific motifs, 86SNPRSV91 and 190A191G206I210E, whereas the PCV2d strains exhibited the 86SNPLTV91 and 190T191G206I210D motifs. The two PCV2e strains, closely related to PCV2a on the phylogenetic tree, shared the identical 86TNKISI91 motif with PCV2a, consistent with findings from previous research.

In terms of protein length, PCV2d strains typically showed an extension of a lysine residue at the C-terminus, altering the Cap protein length from 234 to 235 amino acids. However, the strains SCABTC-PCV2-DW and SCABTC-PCV2-ZB, although classified as PCV2d, lacked this additional lysine and maintained a length of 234 amino acids.

Across 39 PCV2 ORF2 sequences analyzed, including two vaccine strains, 77 amino acid positions were observed to mutate. Notable mutation sites for PCV2e included N77D, L80V, S86T, P88K, L89I, V91I, N232K; for PCV2b, they were V57I, L89R, T/S190A, D210E; and for PCV2d, the mutations were F53I, S90T, T134N, N215I. The ORF2 gene of PCV2 features four highly variable regions: amino acids 53–91, 130–134, 185–191, and 200–217, with additional variability at positions 8, 21, 47, 121, 151, 169, and 232.

Unique mutations relative to reference strains were also detected in some strains during this analysis. For instance, SCABTC-PCV2-NJB exhibited a R10K mutation and a R116G mutation. SCABTC-PCV2-PJA displayed mutations at G117R and N143S, while SCABTC-PCV2-ZB had mutations at V193I and G197S. SCABTC-PCV2-PJG showed a M217L mutation. SCABTC-PCV2-PJA and SCABTC-PCV2-PJB collectively presented mutations at N/A68S, I76L, V123I, T131M, A133V, N134P, and L136Q. A recurrent mutation, R169G, was found in multiple strains, including SCABTC-PCV2-FEI, SCABTC-PCV2-JLQ, SCABTC-PCV2-NJA, SCABTC-PCV2-PJG, SCABTC-PCV2-XWD, SCABTC-PCV2-YQL, and SCABTC-PCV2-ZB. These findings align with previous reports and contribute to the understanding of the molecular diversity within PCV2.

### 3.4 PEDV sequencing and phylogenetic analysis

In this study, we conducted sequencing of the S genes from 16 PEDV strains. The lengths of the S genes varied: SCABTC-PEDV-HAI and SCABTC-PEDV-PG were each 4,149 bp, SCABTC-PEDV-SL was 4,158 bp, and the remaining PEDV S genes measured 4,161 bp. The homology among these 16 PEDV S genes ranged from 95.7 to 99.9%, with their amino acid homology varying from 94.9 to 99.7%. When compared to 57 reference sequences, the homology of these 16 PEDV S genes ranged from 93.6 to 99.9%.

For phylogenetic analysis, the 16 PEDV S gene sequences obtained in this experiment were aligned with reference sequences downloaded from GenBank. The information on the PEDV strains obtained in this experiment, along with the reference strains (Table 3). A phylogenetic tree was then constructed using the Maximum Likelihood method employing the GTR + G + I model with 1,000 bootstrap replications, facilitated by the MEGA11 software. The phylogenetic analysis revealed that among the 16 PEDV strains sequenced in this study, 2 belonged to the G1a genotype (12.5%), and the majority, 14 strains, were classified as G2a (87.5%). These results provide valuable insights into the genetic diversity and evolutionary dynamics of PEDV circulating in the population studied.

In this research, the 16 S genome sequences of Porcine Epidemic Diarrhea Virus (PEDV) obtained from the study, along with 57 reference sequences, were aligned for comprehensive analysis (Figure 3). This meticulous approach unveiled 6 recombination events across the 16 PEDV S gene sequences (Table 4).

Specifically, SCABTC-PEDV-HAI was found to have undergone one recombination event. This event showed breakpoints from 50 to 938, involving SCABTC-PEDV-JL as the major parent and KJ960178 as the minor parent. Further recombination events were detected in SCABTC-PEDV-M6, SCABTC-PEDV-M7, and SCABTC-PEDV-PG. These events shared similar parental backgrounds, with both the major and minor parents being SCABTC-PEDV-SL and JX070671, and occurred over the breakpoints at 2032–4077, 2,125–4,110, and 2,113–4,109, respectively.

SCABTC-PEDV-SL was involved in a recombination event with breakpoints at 1938–4119, where the major parent was KU252649 and the minor parent was JX435305. SCABTC-PEDV-ZYC experienced a recombination event with breakpoints at positions 106–1,090, involving JX435305 as the major parent and KJ645697 as the minor parent.

The observation of multiple recombination events within the same genomic regions suggests a higher propensity for recombination within these specific areas of the PEDV genome. This is indicative of a dynamic genomic landscape where different parental influences repeatedly interact within the same loci, underscoring the complex evolutionary patterns of PEDV.

### 3.5 Comparative analysis of amino acid sequences of PEDV S protein

In this study, we analyzed the amino acid sequences of the PEDV S protein, focusing particularly on the five major neutralizing epitopes, including those in the vaccine strains JQ023161 and AF353511 (Figure 4). These epitopes are identified as S10 (aa 20–220), COE (aa 499–638), SS2 (aa 748–755), SS6 (aa 764–771), and ZC10 (aa 1,368–1,374)^12^. The 16 PEDV S proteins obtained from the experiment showed 60 amino acid mutations within the antigenic epitope S10 (aa 20–220). Notably, there were significant variations between the G1 and G2 genotypes in this epitope.

For the G1 genotype compared to the G2, mutations included alterations such as 27SAN29 to 27CLT29, the deletion of four positions at 58NCGV61, substitution from 62NST64 to 58SSS60, and changes from 68AGCHP72 to 64GTGLE68. Other notable modifications were 84HIRGGH89 to 80YIDSGC85, 177TNAT120 to 113IGAV116, 130SI131 to 126DN127, and 137PTANNDVTT144 to 132PSSGVTS138 with two positions missing. There were also insertions, such as 159SEHSVV164 changing to 153CDGKNVVI160, and point mutations like S177 to A173, F185 to L181, K195 to R191, 199SGG201 to 195KRS197, and C209 to T205.

The COE epitope exhibited a total of eight amino acid mutations, while the SS2 epitope showed no mutations. The SS6 epitope had only one mutation, with the 766th amino acid in SCABTC-PEDV-ZYC changing from proline (P) to leucine (L). No mutations were observed in the ZC10 epitope.

Unique mutations compared to reference strains were identified in the strains obtained in this experiment. These unique mutations varied across different strains, reflecting the diversity and dynamic evolution within the PEDV population. For instance, mutations ranged from simple substitutions like L19R, H71T, and P72S to more complex alterations involving multiple amino acids such as T113I, N114G, T116V, A133S, and many others across different sites and strains. Notably, common mutations such as P229L, F539L, and L998M appeared frequently across several strains, indicating potential sites of evolutionary pressure or functional significance.

This detailed comparative analysis of amino acid sequences highlights the complexity and variability within the PEDV S protein, particularly in its response to immune pressure and vaccine strategies, providing crucial insights into the molecular basis of its antigenic properties.

## 4 Discussion

This discussion explores the historical and current epidemiological trends of Porcine Circovirus Type 2 (PCV2) and Porcine Epidemic Diarrhea Virus (PEDV), which are significant pathogens in the swine industry.

Between January 2023 and July 2024, we tested intestinal samples from 352 diarrheal piglets in Sichuan Province, China, for Porcine Circovirus Type 2 (PCV2) and Porcine Epidemic Diarrhea Virus (PEDV). Sichuan Province is a major pig-producing region in China, making it a statistically significant choice for this study. The research covers 63 pig farms across 17 cities, adequately reflecting the differences in climate, farming practices, and pig density, ensuring the representativeness of the sample. This study aims to cover major pig farms as much as possible, analyzing the impact of environmental and management differences on disease prevalence, thereby providing insights into regional disease patterns to support more effective control measures. A stratified random sampling method was used, with sample sizes reasonably allocated based on farming scale and density in different districts, ensuring that pig herds from various regions had the opportunity to be sampled, thus enhancing the external validity of the results. Farms were randomly selected to avoid human bias and ensure the representativeness of large, medium, and small-scale farms. Through dynamic adjustment of sample sizes, the study ensured sufficient sampling for large-scale farms while preventing oversampling of small farms, optimizing resource allocation and reducing statistical error. Piglets with symptoms of diarrhea were screened as candidate groups, with a focus on detecting PCV2 and PEDV to enhance detection rates and experimental sensitivity. A systematic random sampling method was employed to ensure the randomness and scientific rigor of the sample selection, minimizing bias.

The results showed that 42.33% of the samples (149/352) were positive for PCV2, 50.28% (177/352) were positive for PEDV, and 27.56% (97/352) exhibited co-infections of PCV2 and PEDV. Seasonal analysis of the data indicated higher PEDV infection rates during the winter months, whereas PCV2 infection rates did not show significant seasonal variation. Compared to co-infections of PCV2 and PEDV in other provinces of China, a broader survey in Shandong Province involving 1,325 pig tissue samples revealed an average positivity rate of 36.98% for PCV2 and a co-infection rate with PEDV of 3.47% (2). Another set of tests on 76 severe diarrheal piglet intestinal samples from Henan Province showed higher infection rates, with 82.89% for PCV2 and 68.42% for PEDV, along with a co-infection rate of 57.89% (1).

The survey results highlight significant regional variations in the positivity rates of PCV2 and PEDV among piglets. The high detection rates for both PCV2 and PEDV in diarrheal samples, particularly with a tendency toward co-infection, underscore the challenge these pathogens pose to the swine industry. Co-infections can exacerbate the severity of diarrhea in piglets, leading to more serious impacts on animal health and farm productivity. This study emphasizes the need for targeted interventions and continuous monitoring to manage the spread of these viruses effectively.

This study has identified PCV2d as the predominant genotype of Porcine Circovirus Type 2 (PCV2) in Sichuan Province. Among the 14 PCV2 sequences analyzed, four demonstrated recombination events, each involving at least one parent strain of a different genotype. Such recombination across genotypes facilitates rapid genetic variation in the virus, potentially endowing it with new adaptive features such as enhanced transmissibility, altered pathogenicity, or expanded host ranges.

The PCV2 Cap protein, characterized in this experiment, displayed four highly variable regions: amino acids 53–91, 130–134, 185–191, and 200–217, in addition to several highly variable positions including 8, 21, 47, 121, 151, 169, and 232. These findings align with previous research (27). Notably, the nuclear localization signal (NLS) at the N-terminus of the PCV2 Cap protein functions as a cell-penetrating peptide (CPP), facilitating viral entry into cells. Mutation at the 8th amino acid position from tyrosine (Y) to phenylalanine (F), both of which contain a large aromatic ring, does not likely impact the NLS’s ability to penetrate cells due to their similar structural features (28).

Further complexity in the PCV2 genome is observed in its antigenic sites, which exhibit high variability. The EF loop (residues 134–139) on the capsid protein of PCV2 has been identified as the main specific neutralizing epitope of PCV2. In addition to the EF loop, several other surface loop regions of the PCV2 capsid protein are also of significant immunological importance, including the BC loop (residues 58–66), CD loop (residues 79–94), DE loop (residues 108–116), FG loop (residues 153–156), as well as the GH loop (residues 162–193) and HI loop (residues 204–208) (29). These loop structures are located on the surface of the PCV2 capsid protein, exposed and easily recognized by antibodies. The primary function of these antigenic sites is to induce specific immune responses, particularly by promoting the production of neutralizing antibodies against PCV2. The recognition and binding of these antigenic epitopes are of great importance for the development of PCV2 vaccines and the improvement of diagnostic methods. The high variability at these sites may enhance the virus’s ability to evade host immune defenses, facilitating its continued circulation among diverse hosts or host populations.

Additionally, PCV2 has demonstrated the capability to infect and replicate in mice, suggesting that rodents may serve as alternative hosts or mechanical vectors. Evidence indicates that PCV2 is present in rodents within pig farms but not in those outside such environments, underscoring the potential role of rodents in the virus’s epidemiology (30).

The high mutation rate at antigenic sites complicates the control of PCV2 by potentially reducing vaccine efficacy and increasing the challenge of managing this virus. Despite being a single-stranded DNA virus, PCV2 shows evolutionary dynamism comparable to single-stranded RNA viruses. Therefore, control strategies for PCV2 should emphasize the continuous monitoring of viral genetic variations to effectively manage and mitigate its impact on swine health and production (31). This approach is crucial for developing robust strategies against the rapid evolution and widespread impact of PCV2 in the swine industry.

In this study, we have identified that 87.5% of the Porcine Epidemic Diarrhea Virus (PEDV) strains belong to the G2a genotype, and 12.5% to G1a, aligning with previous findings that G2 (variant strains) is the predominant PEDV genotype circulating in China (32). Among the 16 analyzed PEDV sequences, 7 strains exhibited recombination, leading to a total of 11 recombination events. This indicates a high level of genetic diversity and interaction among different PEDV strains, which can significantly influence the evolutionary capacity of the virus.

The spike (S) protein of PEDV, a major focus of vaccine development and research into virus pathogenicity due to its role as the primary target for host immune responses, contains several critical neutralizing epitopes (33). These include the CO-26 K equivalent (COE) structural domain (499-638aa), SS2 (748-755aa), SS6 (764-771aa), 2C10 (1368-1374aa), and the newly identified S10 epitope. The COE is a critical neutralizing epitope, and antibodies targeting it can effectively block viral infection, making it a key target for vaccine and diagnostic reagent development. SS2 and SS6 are closely related to the membrane fusion function of the S2 protein, and antibodies against these sites can prevent viral fusion with the host membrane, possessing potential neutralizing capabilities. Although 2C10 is not a major neutralizing epitope, as a target for polyclonal antibody binding, it may play a role in enhancing the immune response (34). Notably, the S10 epitope is located within the N-terminal domain (NTD) of the S1 subunit (20–220 aa) and is considered a potential sialic acid-binding domain (35).

Further amino acid alignment analysis of the five major neutralizing epitopes in the PEDV spike protein revealed that only the S10 epitope exhibits a high mutation rate. In contrast, the other epitopes show relatively lower mutation rates. This pattern suggests significant antigenic drift in the PEDV spike protein (36). The variations observed in the PEDV S protein, particularly affecting potential linear B-cell epitopes (8), may enable the virus to effectively evade the host’s immune response at specific antigenic sites. Such evasion mechanisms highlight the adaptive strategies of PEDV, complicating efforts to develop effective vaccines and control measures against this pathogen. These findings underscore the complexity of PEDV’s interaction with the host immune system and the continuous need for detailed genetic and antigenic characterization of this virus to inform ongoing and future vaccine development strategies.

This study investigated the prevalence and genetic diversity of Porcine Circovirus Type 2 (PCV2) and Porcine Epidemic Diarrhea Virus (PEDV) in 352 intestinal samples from diarrheal piglets across Sichuan Province, China. The analysis revealed a significant prevalence of both viruses: 42.33% (149/352) of the samples tested positive for PCV2, 50.28% (177/352) for PEDV, and 27.56% (97/352) exhibited co-infections of both viruses.

Further genetic analysis of the viruses isolated from these samples showed diversity in their genotypic distribution. Of the 14 PCV2 strains analyzed, 2 were identified as belonging to the PCV2b genotype (14.29%), 10 to the PCV2d genotype (71.43%), and 2 to the PCV2e genotype (14.29%). For the 16 PEDV strains detected, 2 were classified as G1a (12.5%), and the majority, 14 strains, were identified as G2a (87.5%). This indicates that the dominant genotype of PCV2 in the region is PCV2d, while for PEDV, it is G2a.

Amino acid sequence comparison between the PCV2 and PEDV strains demonstrated numerous mutations in their antigenic sites. These mutations potentially enable the viruses to evade the immunity provided by current vaccines, underscoring the necessity for ongoing vaccine development and adaptation.

The findings from this study not only highlight the widespread prevalence of PCV2 and PEDV among piglets in Sichuan Province but also provide a detailed genetic and evolutionary analysis of these pathogens. This research offers critical insights into the epidemiology of PCV2 and PEDV in this region, providing valuable information for the development of effective control and prevention strategies. The detailed understanding of the genetic diversity and evolution of these viruses is essential for informing future vaccine design and implementation strategies, aiming to mitigate the impact of these diseases on the swine industry.

## Data Availability

The datasets presented in this study can be found in online repositories. The names of the repository/repositories and accession number(s) can be found in the article/supplementary material.
